# The effects of a (poly)phenol‐rich food intervention on markers of exercise‐induced inflammation and oxidative stress: A randomised controlled trial

**DOI:** 10.1113/EP093383

**Published:** 2026-01-23

**Authors:** Abrar Al Hebshi, Josh Thorley, Ana Rodriguez‐Mateos, Zicheng Zhang, Lewis J James, Tom Clifford

**Affiliations:** ^1^ Department of Clinical Nutrition, Faculty of Applied Medical Sciences Umm Al‐Qura University Makkah Saudi Arabia; ^2^ School of Sport, Exercise and Health Sciences Loughborough University Loughborough UK; ^3^ School of Medicine University of Nottingham Nottingham UK; ^4^ Department of Nutritional Sciences, School of Life Course and Population Sciences King's College London London UK; ^5^ NIHR Leicester Biomedical Research Centre University Hospitals of Leicester NHS Trust and the University of Leicester Leicester UK

**Keywords:** exercise recovery, nutraceuticals, oxidative stress, phytochemicals

## Abstract

This study examined whether consuming a (poly)phenol‐rich food before strenuous muscle‐damaging exercise can modify post‐exercise markers of inflammation and oxidative stress. Using a double‐blinded, randomised, placebo‐controlled, between‐subjects design, 26 recreationally active males (*n* = 15) and females (*n* = 11) consumed higher‐(poly)phenol (H‐POL) foods (dates, dark chocolate, pomegranate; 285.1 mg/day) or lower‐(poly)phenol foods (L‐POL) (cereal bar, milk chocolate, sports drink; 88.3 mg/day) for 3 days before, and then 30 min before, strenuous exercise (100 drop jumps, 50 squat jumps). A range of blood markers associated with inflammation (total and differential leukocytes, interleukin (IL)‐6, IL‐10, IL‐1β, IL‐2, IL‐4, IL‐12, tumour necrosis factor‐alpha (TNF‐α), interferon‐gamma (IFN‐γ), monocyte chemoattractant protein‐1 (MCP‐1), granulocyte colony‐stimulating factor), and oxidative stress (glutathione peroxidase (GPX), 4‐hydroxynonenal (4‐HNE), and urinary 8‐hydroxy‐2′‐deoxyguanosine (8‐OHdG)) were quantified pre, immediately post, 1 and 2 h post‐exercise. One hundred and nineteen plasma (poly)phenol metabolites were measured pre, immediately post and 1 h post‐exercise. Total plasma (poly)phenol concentrations were greater in the H‐POL vs. L‐POL intervention, peaking 1 h post‐exercise (H‐POL: 239.5 ± 87.8 µM vs. L‐POL 58.9 ± 33.8 µM; *P* < 0.001). There were interaction effects for IL‐10 and TNF‐α but no differences with *post hoc* tests. Urinary 8‐OHdG excretion was higher in H‐POL vs L‐POL (condition effect; *P* < 0.001), whereas erythrocytes GPX activity was higher in the L‐POL vs. H‐POL (condition effect; *P* < 0.001). A (poly)phenol food intervention before exercise increased total plasma (poly)phenol concentrations but had limited and inconsistent effects on markers of inflammation and oxidative stress in the 2 h after strenuous exercise.

## Abstract (highlights)

- **What is the central question of this study?**
  (Poly)phenol supplements have antioxidant and anti‐inflammatory effects and may therefore modulate post‐exercise inflammatory responses: do (poly)phenol‐rich foods modify markers of inflammation and oxidative stress following strenuous exercise?
- **What is the main finding and its importance?**
  A higher (poly)phenol meal increased total plasma (poly)phenol metabolites but had limited and inconsistent effects on markers of inflammation and oxidative stress in the 2 h following strenuous exercise. Our findings suggest that acute intake of (poly)phenol‐rich foods before exercise is not sufficient to modify post‐exercise inflammation.

## 1 INTRODUCTION

Athletes often consume supplements with antioxidant and/or anti‐inflammatory effects to modify oxidative stress and inflammation after exercise (Clifford & Howatson, 2022). This is based on the idea that oxidative stress and perturbations to immune function may evoke skeletal muscle damage and delay recovery, contribute to the development of overtraining syndrome, and/or increase the risk of illness (Cheng et al., 2020; Clifford & Howatson, 2022; Kruk et al., 2022; Pingitore et al., 2015). Nonetheless, several studies suggest that supplementation with large doses of individual dietary antioxidants (e.g., vitamin C) or anti‐inflammatory medications (e.g., non‐steroidal anti‐inflammatory drugs; NSAIDs) may impair exercise adaptations such as muscle hypertrophy (Gomez‐Cabrera et al., 2008; Lundberg & Howatson, 2018; Palareti et al., 2016; Paulsen et al., 2014). Therefore, researchers and athletes are increasingly interested in the effects of supplements derived from fruits and vegetables that are rich in (poly)phenols. Indeed, it has been suggested that (poly)phenol supplements may have similar biological effects to dietary antioxidants without interfering with adaptive processes, although findings are equivocal and this remains a controversial topic (Bowtell & Kelly, 2019; Clifford & Howatson, 2022; Martinez‐Negrin et al., 2022)

(Poly)phenols are chemical compounds that contain one or more phenolic rings (Frank et al., 2020). Numerous studies have shown that these compounds have anti‐inflammatory effects in vitro and in animals (Eder et al., 2025; Magrone et al., 2019). In humans, the results are more equivocal, partly due to the low bioavailability of many (poly)phenols (Mukherjee et al., 2022; Ren et al., 2016). Given the low concentrations they reach in vivo, their biological effects are at least partly indirect; indeed, they are unlikely to directly scavenge reactive oxygen species (ROS), but they may upregulate the transcription factor nuclear factor erythroid 2‐related factor 2 (Nrf2) which regulates the endogenous antioxidant defence system (Clifford et al., 2021a; Forman et al., 2014) and interferes with pro‐inflammatory pathways, such as the nuclear factor kappa‐light‐chain‐enhancer of activated B cells (NF‐κB) pathway (Kobayashi et al., 2016; Xie et al., 2017). Downstream, this can dampen the release of pro‐inflammatory cytokines and chemoattractants that may amplify the inflammatory response (Granica et al., 2015).

Most studies examining the anti‐inflammatory effects of (poly)phenols after exercise have provided concentrated supplements derived from turmeric, pomegranate, green tea, cherries, blueberries or beetroot, which tend to contain high amounts of a specific (poly)phenol class (e.g., anthocyanins) but only a limited range of (poly)phenols (Bell et al., 2016; Clifford et al., 2017; McFarlin et al., 2016; Thorley et al., 2023). Indeed, there is limited research on the effects of (poly)phenol‐rich food sources, consumed as a diet or meal, on exercise‐induced inflammation. In one study, Koivisto et al. (2019) found that providing several antioxidant‐rich foods (∼21.2 mmol/day of antioxidants), including dark chocolate, cranberries and fruit smoothies, significantly increased the ferric reducing ability of plasma, a measure of antioxidant capacity, and reduced C‐reactive protein (CRP) and interleukin (IL)‐13 and ‐6 in elite athletes over a 3‐week training camp. However, there are no studies examining the effects of a shorter‐term (<5 days) (poly)phenol‐rich food intervention on markers of exercise‐induced inflammation or oxidative stress, a dosing strategy that more closely replicates post‐exercise nutrition practices (Clifford & Howatson, 2022). In addition, no studies to date have examined the effects of (poly)phenol‐rich meals before exercise known to induce muscle damage and evoke soreness and decreases in muscle function, symptoms which (poly)phenols are proposed to alleviate via their anti‐inflammatory effects (Bowtell & Kelly, 2019; Clifford & Howatson, 2022). Finally, most studies with (poly)phenols measure biological markers several hours or days post‐exercise (Rickards et al., 2021), when the acute systemic inflammatory response has largely dissipated (Paulsen et al., 2012) and the bioactive ingredients in the interventions have been excreted, which may limit the ability to detect true effects.

Meals or a diet rich in (poly)phenols could offer several advantages over concentrated supplements. Firstly, they could have greater bioavailability and bioactivity than concentrated supplements because they contain a more diverse range of (poly)phenol and non‐(poly)phenol compounds, increasing the opportunity for additive or synergistic interactions (Bohn, 2014). In addition, albeit the evidence is limited (Martinez‐Negrin et al., 2022), there remains concern that high doses of concentrated (poly)phenol supplements could impair exercise training adaptations (Schwarz et al., 2018; Scribbans et al., 2014) or evoke deleterious pro‐oxidant effects. Finally, many (poly)phenol‐based supplements have not been tested for prohibited substances, meaning they may not be suitable for athletes subjected to regular drug tests for banned substances. Consequently, the aim of this study was to examine whether consuming a (poly)phenol‐rich meal in the days before and morning before strenuous muscle damaging exercise can modify subsequent changes in markers of inflammation and oxidative stress. We hypothesised that the (poly)phenol intervention would modulate markers of oxidative stress and inflammation after exercise.

## 2 METHODS

### 2.1 Ethical approval

This study and the protocol were approved by the Loughborough University Ethics Committee (2022‐7234‐9300). All participants gave written informed consent prior to participation and the research was conducted in accordance with the Declaration of Helsinki. The study was conducted at the National Centre for Sport and Exercise Medicine in Loughborough, UK, between June 2022 and August 2023 and the methods were preregistered on the Open Science Framework (https://osf.io/nxsep) prior to data collection.

### 2.2 Participants

**FIGURE 1:**
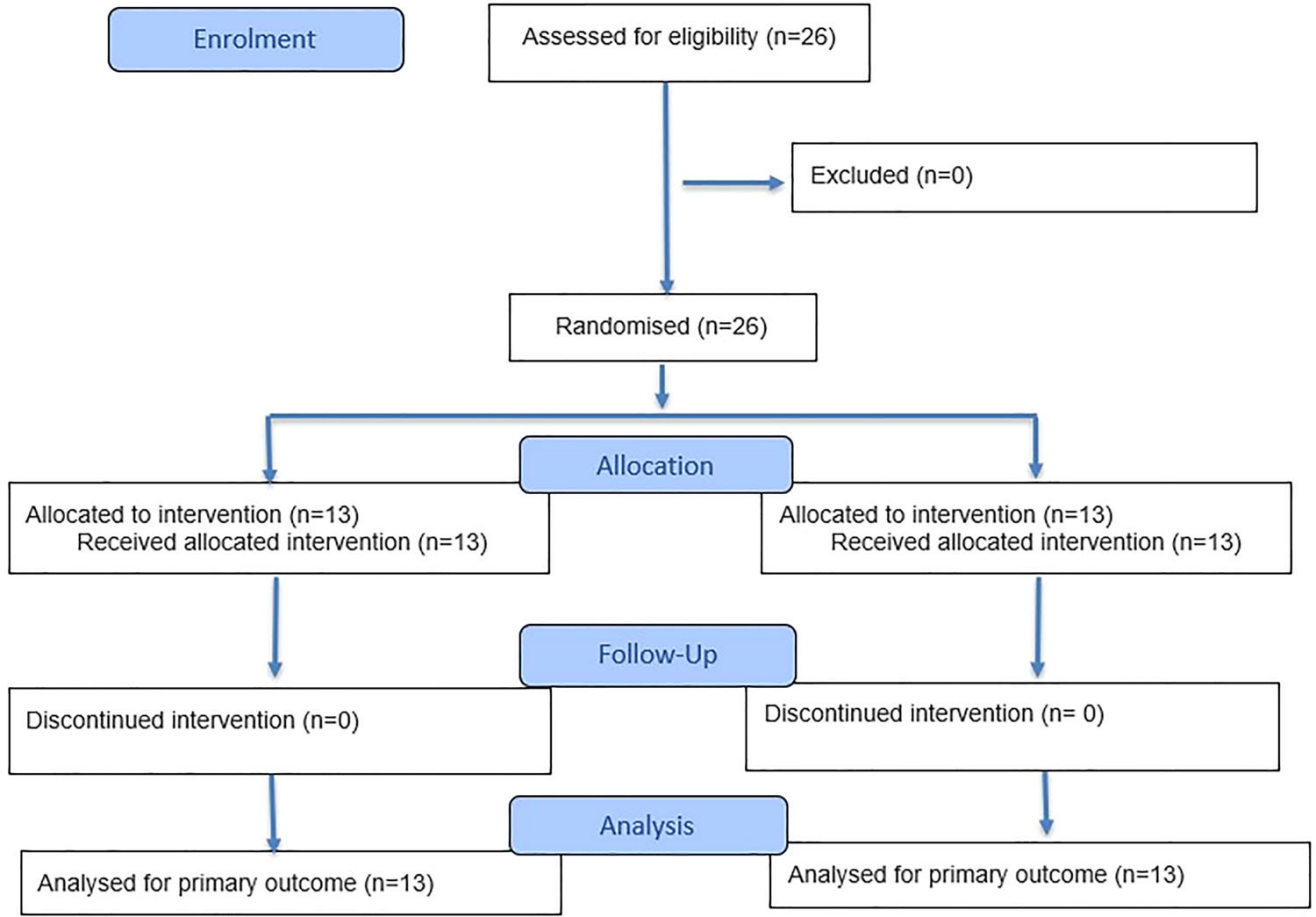
Participant flow diagram, enrolment to analysis.

Twenty‐six healthy males (*n* = 15) and females participated in this study. There were six females in the intervention condition and five females in the control condition. Of the 11 females, seven were normally menstruating for at least 5 months without the use of any hormonal contraceptives, and four were using oral and intrauterine contraceptives. Participant physical characteristics are presented in Table 1. Testing was scheduled during the early follicular phase, when oestrogen is more stable and less likely to influence the study outcomes (Oosthuyse & Bosch, 2010). For study inclusion, participants had to be 18–40 years old and defined as recreationally active (regularly exercising 2–4 days/week), but performing no more than one lower body resistance training session per week in the past 2 months. All volunteers completed a health screening questionnaire and were included if they did not meet one or more of the following exclusion criteria: (1) sedentary or highly trained individuals (completing >4 resistance training sessions per week in the past 6 months); (2) had a food allergy or intolerance; (3) had medical history of a chronic disease (respiratory, renal, cardiovascular, gastrointestinal, or metabolic disease); (4) had a previous musculoskeletal injury or acute ankle/knee pain in the last 6 months that contraindicates with the study procedures; (5) were taking prescribed anti‐inflammatory/antioxidant medications <1 month prior to participation, (6) following a specialised dietary regimen such as Mediterranean, vegetarian or DASH diets. No participants were excluded after screening; a participant flow diagram of study enrolment is presented in Figure 1.

**TABLE 1:** Participants’ physical characteristics and exercise workload in the high‐(poly)phenol (H‐POL) and low‐(poly)phenol (L‐POL) conditions.

	H‐POL	L‐POL	*P*
Physical characteristics			
Age (years)	24 ± 4	24 ± 5	0.968
Stature (cm)	173 ± 7	174 ± 9	0.695
Body mass (kg)	68 ± 9	72 ± 13	0.439
Sex (male/female)	7/6	8/5	
Exercise workload			
Mean DJ height (cm)	25 ± 7	26 ± 6	0.757
Max DJ height (cm)	28 ± 6	29 ± 9	0.851
% difference from max	−13%	−9%	0.539
Mean SJ height (cm)	22 ± 7	23 ± 7	0.959
Max SJ height (cm)	29 ± 7	29 ± 5	0.911
% difference from max	−23%	−22%	0.840

Values are means ± SD. *n* = 13 per condition. D, drop jump; SJ, squat jump. Analysis performed with Student's independent *t*‐test.

### 2.3 Experimental design

**FIGURE 2:**
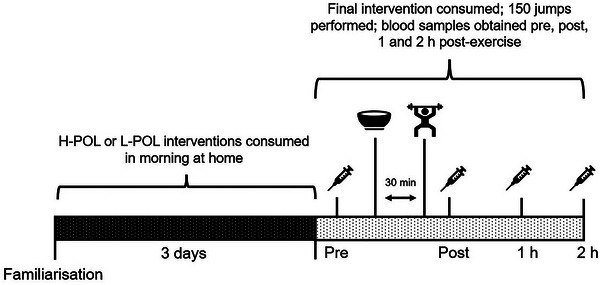
Schematic outline of study procedures. After a familiarisation visit and randomisation, interventions (higher (poly)phenol (H‐POL) or lower (poly)phenol (L‐POL)) were consumed for 3 days before participants attended the lab on day 4. Pre‐exercise (Pre) blood samples were taken fasted; the final intervention was consumed 30 min before exercise (150 jumps). Further blood samples were obtained immediately post‐exercise (Post) and 1 and 2 h post‐exercise.

A randomised, double‐blind, placebo‐controlled, between‐subjects design was used for this study. A schematic illustration of study procedures is presented in Figure 2. Participants attended the laboratory twice; first for a familiarisation visit, and second for the experimental trial. During the familiarisation visit, stature and body mass were recorded, and participants were given a demonstration of the exercise protocol. Their maximum drop and squat jump height were then recorded, using the methods described below. Each participant was then randomly assigned to one of the two conditions (higher‐(poly)phenol (H‐POL) vs lower‐(poly)phenol (L‐POL)) by a member of the study team not involved in data collection using minimisation. For minimisation, sex and countermovement jump (CMJ) height were used as prognostic factors, whereby participants were assigned to each intervention based on these factors at study entry. This was preferred to simple randomisation as it enabled us to better balance the participants’ physical characteristics across the interventions. Participants were then provided with an opaque food bag containing their interventions with instructions to consume them for 3 days before the main exercise trial, each morning as a breakfast. We chose a 3‐day intake of (poly)phenols based on previous recommendations that a preload of 3 days or more is better than an acute single dose, but we reasoned that longer than 3 days before the main trial could become burdensome and affect participant compliance (Bowtell & Kelly, 2019).

For the experimental trial, participants arrived at the laboratory after a 10–12 h overnight fast. Upon arrival, resting blood and urine samples were collected, and their final dietary intervention was consumed. After a 30‐min rest, participants completed the exercise protocol (described below). Further blood and urine samples were collected immediately post, 1 h and 2 h after exercise, while the participant rested in the laboratory and only water was allowed (ad libitum).

### 2.4 Dietary interventions

Participants assigned to the H‐POL condition consumed dark chocolate (70% cocoa), dried dates and pomegranate juice; in the L‐POL diet, they consumed milk chocolate, a granola bar and a sports drink. The composition of the diets is provided in Table 2. These specific foods were initially selected based on previous research detailing their antioxidant (Carlsen et al., 2010) and (poly)phenol content (http://phenol‐explorer.eu/) as well as the ability of the lower and higher (poly)phenol foods to be matched for macronutrient and energy content. The H‐POL foods were rich in flavonols, especially proanthocyanidins, ellagitannins and anthocyanins. All foods were commercially available in the UK. The total (poly)phenol content of the foods was then analysed independently to confirm the H‐POL condition provided more (poly)phenols. The list of (poly)phenols measured is given in Appendix Table A1, and the methods used for this analysis are provided in the Appendix. Because the control group was shared with another study examining the effects of a capsule supplement (https://osf.io/3k6yp), participants in both groups also consumed two capsules containing 500 mg of inulin (Blackburn Distributions, Lancashire, UK), at the same time points as the food, which acted as a placebo control. All data were collected in parallel at the same time points as one large study. This small amount of inulin would not be expected to modify the outcomes and was therefore deemed a suitable control.

**TABLE 2:** Nutritional composition of the lower‐(poly)phenol and higher (poly)phenol conditions.

	Lower (poly)phenol				Higher (poly)phenol			
	Milk chocolate	Granola bar	Sports drink^*^	Total	70% cocoa chocolate	Ajwa dates	Pomegranate juice	Total
Serving size/day	53 g	30 g	400 mL		50 g	40 g	150 mL	
Energy (kcal)	283	140	96	519	283	143	98	523
Fat (g)	16.0	5.4	0.0	21.4	20.5	0.32	0.0	20.8
Carbohydrate (g)	30.0	19.3	24.0	73.3	17.0	34.4	24.0	75.4
Protein (g)	3.9	2.6	0.0	6.5	4.8	1.2	0.5	6.4
Total (poly)phenols (mg)	79.9	8.3	—	88.3	215.1	13.9	56.1	285.1

* (Poly)phenol content data not available. Antioxidant activity measured as 0.01 mmol/100 g (Carlsen et al., 2010).

### 2.5 Exercise and diet controls

Participants were instructed to avoid engaging in any strenuous exercise 48 h prior to the experimental visit. Participants were encouraged to continue with their habitual dietary intake and not to increase their consumption of naturally occurring high (poly)phenol foods during the 4 days of the trial period. To facilitate this, they were provided with a list of (poly)phenol‐rich foods and beverages (e.g. berries, pomegranate, apple, pears, dates, dark chocolate/cocoa, nuts, turmeric, green or dark tea, red wine and fruit juices). In addition, dietary supplements, such as vitamins, minerals and protein powders, were prohibited during the experimental period. Participants were never explicitly told the aim of the study was to examine (poly)phenol‐rich foods or that the restricted foods were rich in (poly)phenols to prevent them from modifying their diet during the study.

To evaluate compliance, participants were provided with a food weighing scale and diary and asked to record their intake 1 day before the experimental visit. These diaries were subsequently analysed using the nutrition analysis software Nutritics (Research Edition, v5.09, 2019, Nutritics, Dublin, Ireland) for total energy, macronutrient (carbohydrate, protein, fat) content, as well as vitamin C and E (main dietary antioxidants), and omega‐3 fatty acids, which may modulate inflammation post‐exercise (Fernández‐Lázaro et al., 2024).

### 2.6 Exercise protocol

The exercise protocol consisted of 100 drop jumps, then 1 min rest, followed by 50 consecutive squat jumps. The drop jumps were performed from a 0.6 m box in five sets of 20 repetitions, with each jump separated by 5 s, and each set by 1 min of rest. To ensure participants were working maximally, their jump heights were recorded with a jump mat (JumpMat™, FSL Scoreboards, Cookstown, UK). Drop jump or squat jump height (cm) was recorded automatically by the jump mat, which calculates vertical displacement from flight time. Throughout the protocol, participants were encouraged to maintain their jump heights ≤20% of their maximum jump height determined at familiarisation; this was achieved via continuous verbal encouragement. Participants were allowed to drink water ad libitum throughout.

This specific exercise protocol was chosen as previous studies have shown that it stimulates an acute inflammatory response, as evident by increases in total and differential leukocyte counts, and cytokines including IL‐6 (Arazi et al., 2019; Chalari et al., 2019; Chatzinikolaou et al., 2010; Clifford et al., 2016, 2019; Thorley et al., 2023). In addition, as this type of exercise induces significant muscle damage (Clifford et al., 2016; Kamandulis et al., 2022) it is often used as a model to test the effects of (poly)phenol interventions; thus, it was felt the results would be relevant to situations that may affect athlete recovery.

### 2.7 Blood sample analysis

Venous blood samples were collected via venipuncture pre‐exercise, immediately post‐exercise, and 1 h and 2 h post‐exercise. Blood was drawn into a 4 mL vacutainer coated with EDTA and immediately centrifuged at 1500 *g* (4°C) for 10 min, with the supernatant plasma layer then pipetted into cryovials, which were stored at −80°C prior to analysis.

Erythrocyte hemolysates were collected as per previously described methods (Veskoukis et al., 2016). Briefly, following plasma collection and removal from the vacutainer, erythrocyte lysate was aliquoted from all samples. The white buffy coat layer above the erythrocytes was carefully removed, and an equal volume (1:1) of 0.9% NaCl was added to the sample, followed by centrifugation at 1500 *g* for 10 min at 4°C. The saline supernatant was then discarded, and an equal volume (1:1) of distilled water was added to the packed erythrocytes. The sample underwent a final centrifugation at 4000 *g* for 15 min at 4°C. The resulting erythrocyte haemolysate was aliquoted and stored at −80°C for later analysis.

An automated haematology analyser (Horiba cell counter Yumizen H500, Horiba, Ltd, Montpellier, France) was used to measure total circulating platelets, white blood cell (WBC) and differential (neutrophil, monocytes, eosinophils and basophils) leukocyte counts at all time points from whole EDTA blood. The systemic inflammatory index (SII) was calculated by multiplying the neutrophil lymphocyte ratio by platelet counts (Walzik et al., 2021).

Cytokines (IL‐6, IL‐10, TNF‐α, IL‐1β, IL‐12, IL‐2, IFN‐γ, IL‐4), monocyte chemoattractant protein‐1 (MCP) and growth colony stimulating factor (G‐CSF) were all measured using the Simple Plex™ ELLA rapid detection enzyme‐linked immunosorbent assay (ELISA) microfluidics platform (ProteinSimple, Bio‐Techne, Oxford, UK). Briefly, plasma samples were diluted in a 1:1 ratio (2‐fold dilution) with a diluent according to the manufacturer's instructions and transferred into a cartridge inlet. The diluted sample was directed through microfluidic channels containing two glass nano reactors coated with the target capture antibody. Target detection antibodies subsequently migrated to the glass nano reactors, which were then scanned, and analyte concentrations were provided in duplicate or triplicate (MCP‐1 and G‐CSF only due to cartridge size). The intra‐assay coefficients of variation (CV) were between 10.0 and 14.4% for IL‐1β, IL‐2, IL‐4 and IL‐12, but <5% for all other proteins except IL‐10 (7.1%).

Plasma 4‐hydroxynonenal (4‐HNE) was quantified as a biomarker of lipid peroxidation with a commercially available assay kit (cat. no. ab238538, Abcam, Cambridge, UK). 8‐Hydroxy‐2′‐deoxyguanosine (8‐OHdG) was quantified in urine as a biomarker of DNA oxidative damage using a commercially available competitive ELISA kit (cat. no. ab201734, Abcam). Concentrations were corrected for creatinine (cat. no. 500701, Cayman Chemical Co., Ann Arbor, MI, USA). Due to limited space on the ELISA plates, all 8‐OHdG and 4‐HNE analyses were run singly, and therefore the CV was not determined. Glutathione peroxidase (GPX) enzyme activity was measured in erythrocyte lysates with a commercially available ELISA kit (cat. no. 703102, Cayman Chemical Co.). In the analysis, 1 unit of GPX activity was defined as the number of enzymes forming 1 nmol of NAPDH to NADP^+^ per min (nmol/min/mL). All analysis was performed according to the manufacturer's instructions. The intra‐assay CV for GPX analysis was 1.6%.

### 2.8 Quantification for (poly)phenol metabolites

Quantification of (poly)phenols as well as their metabolites in plasma samples was performed based on the method previously described (Domínguez‐Fernández et al., 2021) using micro‐solid phase extraction (SPE) and UPLC–electrospray ionisation (ESI)–triple quadrupole (QqQ)–tandem mass spectrometry (MS/MS). Briefly, plasma samples were centrifuged at 15,000 *g* (15 min, 4°C), and 350 µL supernatant was collected and acidified with 4% phosphoric acid (1:1, v/v). Six hundred microlitres of the mixture were then loaded onto the Oasis hydrophilic−lipophilic balanced μ‐SPE 96‐well cartridge plate. The plate was washed with water (200 µL) and 0.2% acetic acid (200 µL), and targeted compounds were eluted with 90 µL acidified methanol (0.1% formic acid and 10 nM ammonium formate, v/v) into a 96‐well collection plate. Additional water (35 µL) and internal standards (0.25 mg/mL taxifolin, 5 µL) were fortified in the same collection plate, making a 130 µL final sample volume. Samples on the collection plate were finally transferred into the HPLC vials and stored at −70°C before the analysis.

Quantification of (poly)phenols as well as their metabolites in plasma samples was achieved using a UPLC system (Vanquish, Thermo Fisher Scientific, Runcorn, UK) equipped with electrospray ionisation and triple quadrupole mass spectrometry (Vantage, Thermo Fisher Scientific). Five microlitres of samples was injected into the system and passed through a Raptor Biphenyl reversed phase column (2.1 × 50 mm, 1.8 µm, Restek, Bellefonte, PA, USA) coupled with a compatible guard cartridge (5 × 2.1 mm, 2.7 µm, Restek) under 30°C. Mobile phases were HPLC grade water and acetonitrile that were both acidified with 0.1% formic acid (v/v), as solvent A and B, respectively. The gradients were as followed: 0–1 min, 1% B; 1–4 min, 1–12% B; 4–8 min, 12% B; 8–11 min, 12–15% B; 11–11.5 min, 15–30% B; 11.5–12 min, 30–99% B; 12–14 min, 99% B; 14–14.1 min, 99–1% B; and 14.1–16 min, 1% B, with a flow rate of 0.35 mL/min. The MS analysis was performed under the following conditions: collision gas pressure 1.0 mTorr, capillary temperature 270°C, vaporizer temperature 350°C, sheath gas pressure 49 arb, aux gas pressure 10 arb, spray voltage 3000 V. All the compounds were analysed in negative ion mode. One hundred and thirty‐four standards were used for the identification and quantification of the compounds. All standards were mixed as the master calibration mixture, and 13 dilutions were made for plotting the calibration curve. Taxifolin (0.25 mg/mL) was fortified into all samples and calibration mixes in the same concentration as the internal standard. The final multiple reaction monitoring (MRM) analysis method was generated with the confirmed retention time of the compounds, 2‐min retention time (RT) window, 0.70 full width at half maximum, quadrupole 1 (FWHM Q1) peak width, and 3.0 s cycle time. The peak integration was conducted with the TraceFinder 5.0 Software (Thermo Fisher Scientific) and data calculation was performed in Microsoft Excel. The area ratios of the target compounds to internal standard taxifolin were used in the quantification to balance the variations in device performance during the batch run.

### 2.9 Statistical analysis

The sample size for this study was determined for changes in IL‐6, using the ANOVA power shiny app (Lakens & Caldwell, 2021). We chose IL‐6 over other biomarkers because not only is it widely measured, but several studies report a significant increase in this marker following similar exercise. A mean between‐condition change and standard deviation of 1.4 pg/mL and 1.0 pg/mL, respectively, was used for our simulation (2000 runs); these inputs were based on the results of comparable studies with similar designs demonstrating a dietary intervention's ability to modify post‐exercise IL‐6 levels (Bell et al., 2016, 2015; Kerasioti et al., 2013). This estimated 12 participants per condition would provide ≥85% power to detect a time, intervention and interaction effect.

All data are expressed as means ± SD; *P* ≤ 0.05 was considered statistically significant. Data were analysed using jamovi 2.4.8Ink (retrieved from https://www.jamovi.org). Normal distribution of data was evaluated by inspecting histograms and Q‐Q plots of the residuals. All data were normally distributed except for IL‐6, IFN‐γ, 8‐OHdG and eosinophils, which displayed slight deviations from normality; however, raw data were used for statistical analysis as the deviations were mild and there were no differences when the data were analysed with log transformations to lessen skewness. Participants’ physical characteristics, average dietary intake and exercise intensity were evaluated using an independent sample Student's *t*‐test.

A linear mixed model (LMM) was performed to examine the effects of the intervention on plasma (poly)phenol metabolites, total and differential WBC counts, inflammatory and oxidative stress markers. As there were some data points missing at random due to four missed blood samples and two missed urine samples, LMM was preferred to the mixed model ANOVA specified in the pre‐registration protocol. Analysis was still performed on *n* = 13 per group for most outcomes, including our primary outcome, IL‐6. Fixed effects in the analysis were condition (L‐POL vs. H‐POL), time and condition × time, with participants as random effects. Models were fit with restricted maximum likelihood (REML). If main LMMs indicated significant time or interaction effects, a Holm–Bonferroni *post hoc* correction was performed to locate the significant differences (Gaetano, 2013). Spearman's rank correlation was performed to explore any relationships between the change in total plasma (poly)phenol concentrations and change in all inflammatory and oxidative stress outcomes. Spearman's ranked correlation was performed as the (poly)phenol data were not normally distributed. Correlations were performed on pre–post and pre–1 h post mean changes for each variable.

Effect sizes for LMM analysis are presented as partial eta squared (η_p_^2^: small 0.01, medium 0.05, large 0.14) (Cohen, 1988). Note, these only explain the variation in the fixed effects and do not account for random effects and thus should be interpreted accordingly. Hedges *g* effect sizes are presented for any significant *post hoc* interaction effects; values of 0.20, 0.50 and 0.80 correspond to small, medium and large effect sizes (Cohen, 1992). Graphs were created by GraphPad Prism (v9.4.1, GraphPad Software, Boston, MA, USA).

## 3 RESULTS

As ≥35% of all samples were below the limits of detection for IL‐1β (0.064 pg/mL), IL‐2 (0.18 pg/mL), IL‐4 (0.16 pg/mL) and IL‐12 (0.16 pg/mL), these data were not included in the final analysis.

### 3.1 Participants physical characteristics and exercise jump heights

All 26 participants completed the study. All participants returned their empty food bags prior to the main trial and confirmed 100% compliance with the interventions. No significant differences were observed for physical characteristics or exercise jump heights between interventions, suggesting that they were well‐matched (Table 1).

### 3.2 Dietary intake

Energy, macronutrient, vitamin C and E, and omega‐3 fatty acid intake for the two conditions are shown in Table 3. There were no significant differences in dietary intake between the two conditions. The 1‐day food record diary showed that participants adhered to the diet instructions before the trial and no adverse events were reported during the trial period.

**TABLE 3:** Participants’ dietary intake 1 day prior to the exercise trial in the higher‐polyphenol (H‐POL) and lower‐(poly)phenol (L‐POL) interventions.

	H‐POL	L‐POL	*P*
Energy (kcal)	1970 ± 1252	1904 ± 494	0.875
Protein (g)	85 ± 52	91 ± 47	0.811
Carbohydrate (g)	233 ± 151	212 ± 82	0.703
Fat (g)	77 ± 54	81 ± 29	0.852
Vitamin C (mg)	58 ± 59	42 ± 42	0.498
Vitamin E (mg)	3.1 ± 1.5	3.7 ± 3.1	0.625
Omega‐3 fatty acid (g)	0.44 ± 0.75	0.92 ± 1.41	0.376

Values are means ± SD. *n* = 13 per condition. Data exclude the interventions. Analysis performed with independent *t*‐tests.

### 3.3 Plasma (poly)phenols concentrations

**FIGURE 3:**
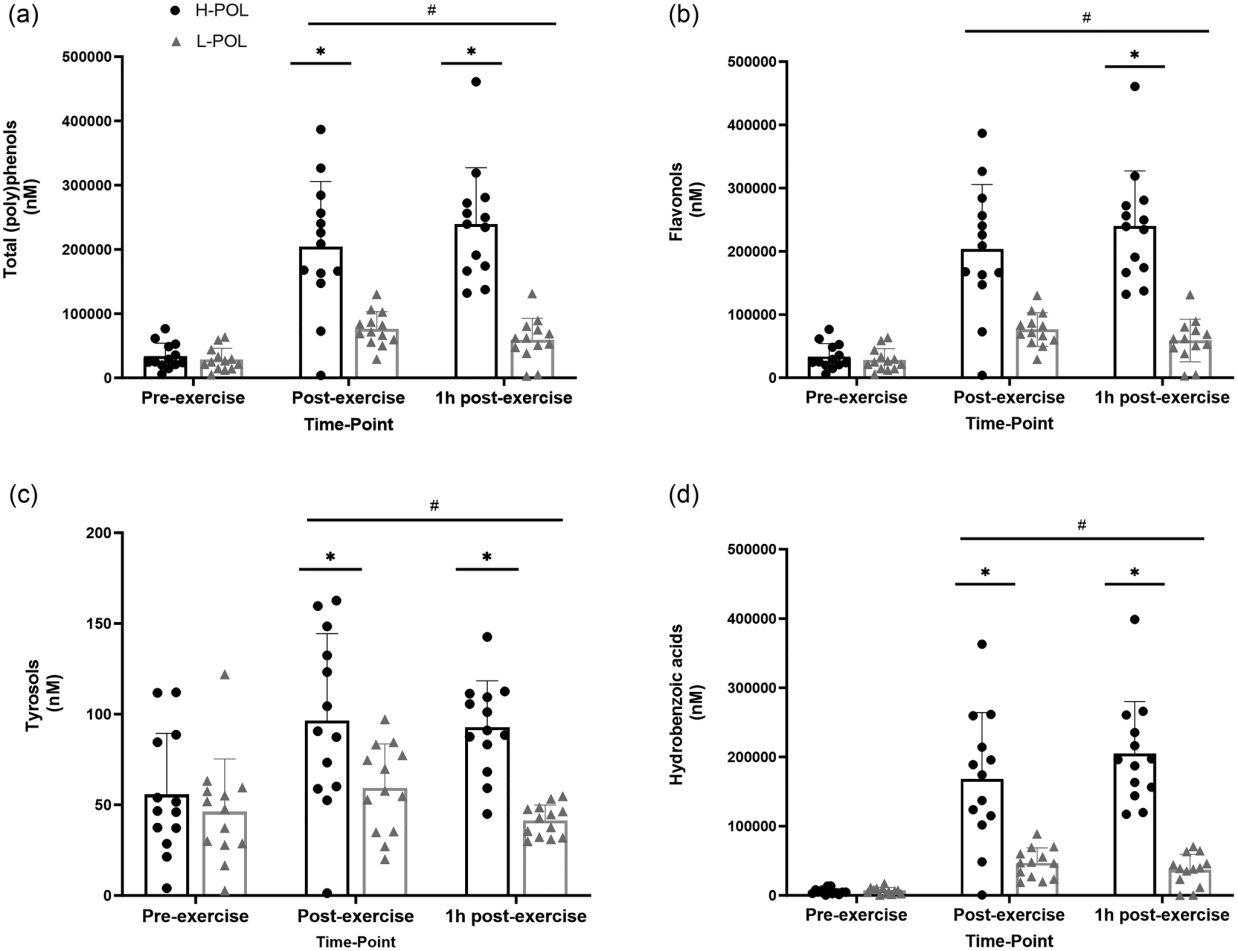
Plasma total (poly)phenols (a), flavonols (b), tyrosols (c), hydrobenzoic acids (d) concentrations at pre‐exercise, post‐exercise and 1 h post exercise (*n* = 13 per condition). Values are expressed as means ± SD. *Significant *post hoc* interaction effects; #significant main effect of time; actual *P*‐values are presented in main text. Each circle represents the higher (poly)phenol condition (H‐POL); each triangle represents the lower (poly)phenol condition (L‐POL). Each circle or triangle represents individual participant data. Analysis performed with linear mixed models.

Plasma concentrations of 119 (poly)phenol metabolites were quantified pre‐exercise, immediately post‐exercise and 1 h post‐exercise (there was insufficient sample to analyse concentrations 2 h post‐exercise). A full list of the (poly)phenols measured is available in Appendix Table A2. Statistical analysis was initially performed on a sum total of the (poly)phenols measured, then each of the 16 (poly)phenol groups displayed in Table A2. There were significant condition and interaction effects for total (poly)phenol concentrations (both *P* < 0.001; η_p_^2^ = 0.67, η_p_^2^ = 0.42, respectively). *Post hoc* tests showed that concentrations were higher in H‐POL immediately post exercise (*P* < 0.001; *g* = 1.65) and 1 h post‐exercise (*P* < 0.001; *g* = 2.62) (Figure 3).

Of the 16 (poly)phenol groupings, flavonols, tyrosols and hydrobenzoic acids were the only groups that showed significant condition (*P* < 0.001, η_p_^2^ = 0.47; *P* = 0.002, η_p_^2^ = 0.34; *P* < 0.001, η_p_^2^ = 0.48, respectively) and interaction (*P* < 0.001, η_p_^2^ = 0.39; *P* = 0.009, η_p_^2^ = 0.18; *P* < 0.001, η_p_^2^ = 0.34, respectively) effects. *Post hoc* tests showed that flavonols, tyrosols and hydrobenzoic acids were greater in H‐POL immediately post‐exercise (all *P* < 0.001; *g* ≥ 0.81) and 1 h post‐exercise (all *P* < 0.001; *g* ≥ 2.03). Note, the analysis for hydroxybenzoic acids violated the homogeneity of variance (Figure 3).

Further analysis revealed that eight individual metabolites showed interaction and/or condition effects: (−)‐epicatechin‐3‐*O*‐sulfate (both *P *< 0.001), tyrosol sulfate sodium salt (both *P* < 0.001), (4*R*)‐5‐(3′,4′‐dihydroxyphenyl)‐γ‐valerolactone‐4′‐*O*‐sulfate sodium salt (interaction; *P* = 0.019), gallic acid (condition *P* = 0.025; interaction *P* = 0.002); protocatechuic acid 3‐*O*‐sulfate (both *P* < 0.001), protocatechuic acid 4‐*O*‐sulfate (both *P* < 0.001), 4‐methylgallic‐3‐*O*‐sulfate (condition *P* < 0.001; interaction *P* = 0.012) and isovanillic acid 3‐*O*‐sulfate (interaction *P* = 0.011). All η_p_^2^ were ≥0.16.

**FIGURE 4:**
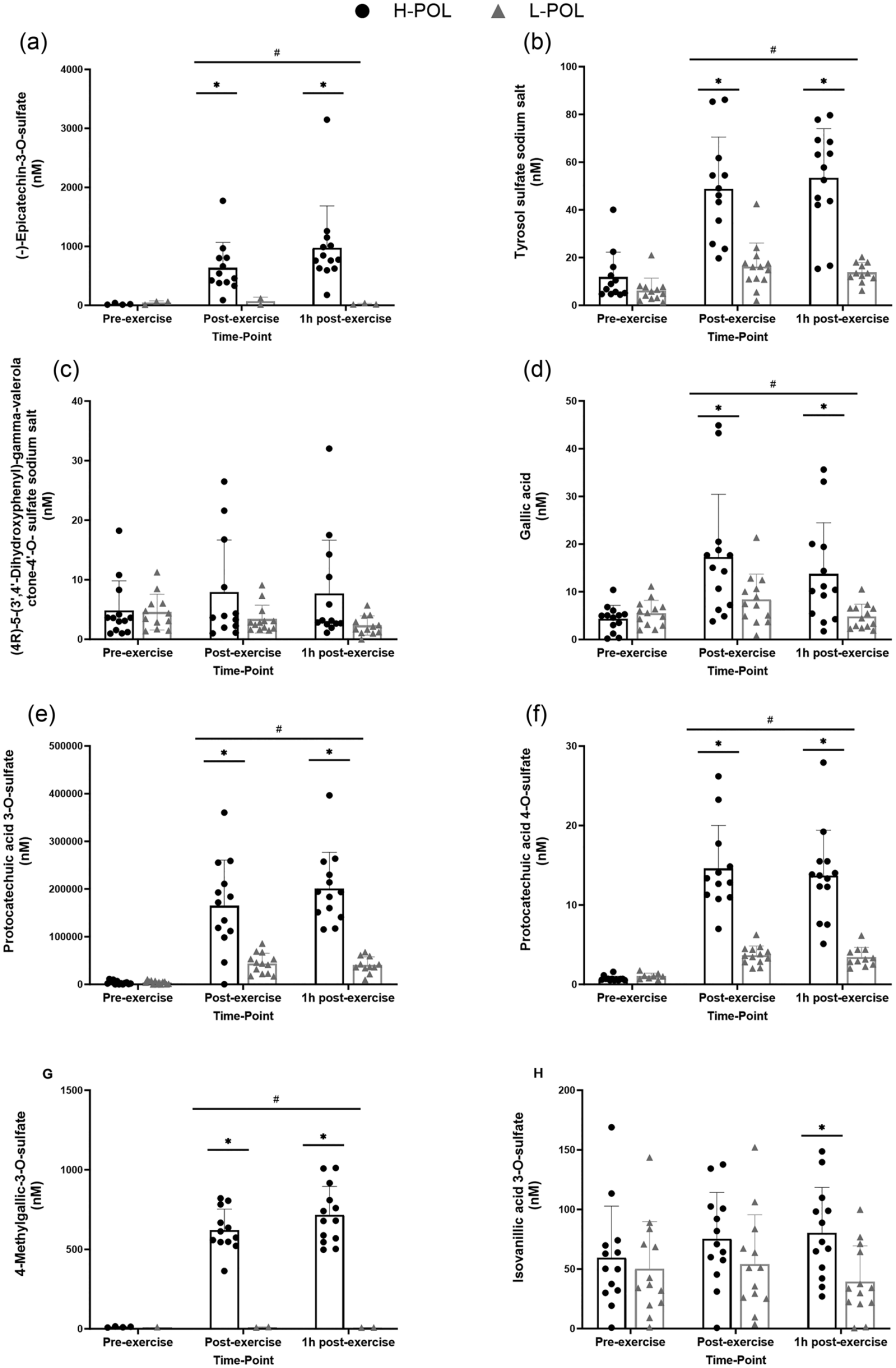
(Poly)phenol metabolites: (−)‐epicatechin‐3‐*O*‐sulfate (a), tyrosol sulfate sodium salt (b), (4*R*)‐5‐(3′,4′‐dihydroxyphenyl)‐γ‐valerolactone‐4′‐*O*‐sulfate sodium salt (c), gallic acid (d), protocatechuic acid 3‐*O*‐sulfate (e), protocatechuic acid 4‐*O*‐sulfate (f), 4‐methylgallic‐3‐*O*‐sulfate (g), and isovanillic acid 3‐*O*‐sulfate (h) concentrations at pre‐exercise, post‐exercise and 1 h post exercise (*n* = 13 per condition). Values are expressed as mean ± SD. *Significant *post hoc* interaction effects; #significant main effect of time. Actual *P*‐values are presented in the main text and Appendix Table A3. Each circle represents the higher (poly)phenol condition (H‐POL); each triangle represents the lower (poly)phenol condition (L‐POL). Each circle or triangle represents individual participant data. Data are *n* ≤ 13 per condition for some metabolites and time points as the metabolite was not detected. Analysis performed with linear mixed models.

*P*‐values for *post hoc* tests are presented in Appendix Table A3 due to their large number. *Post hoc* tests showed that all but (4*R*)‐5‐(3′,4′‐dihydroxyphenyl)‐γ‐valerolactone‐4′‐*O*‐sulfate sodium salt and isovanillic acid 3‐*O*‐sulfate were greater in H‐POL immediately post‐exercise (all effect size *g* ≥ 0.86) and all but (4*R*)‐5‐(3′,4′‐dihydroxyphenyl)‐γ‐valerolactone‐4′‐*O*‐ sulfate sodium were higher in H‐POL 1 h post‐exercise (all effect size *g* ≥ 1.05). (−)‐Epicatechin‐3‐*O*‐sulfate and protocatechuic acid 4‐*O*‐sulfate were analysed after log10‐transformation to reduce skewness. Protocatechuic acid 3‐*O*‐sulfate violated homogeneity of variance, so the results should be interpreted cautiously. Data for these individual metabolites are presented in Figure 4.

Table 4 shows the results of the main LMM analysis for leukocyte counts and associated markers. *Post hoc* tests showed that total leukocyte concentrations increased immediately post‐exercise (*P* < 0.001) and 2 h post‐exercise (*P* < 0.001). Neutrophils increased at all time points post‐exercise (all *P* < 0.001). Monocytes and basophils increased post‐exercise only (both *P* < 0.001). Eosinophils rose immediately post‐exercise (*P* = 0.034) before decreasing below resting values at 1 h (*P* = 0.006) and 2 h (*P* = 0.032) post‐exercise. Platelets increased post‐exercise (*P* < 0.001) and there was a condition effect with concentrations higher in the H‐POL condition. Neutrophil–lymphocyte ratio and SII were both increased by 1 h (*P* < 0.001) and 2 h post‐exercise (*P* < 0.001). PL ratio decreased post‐exercise but was elevated by 1 and 2 h post‐exercise (all *P* < 0.001).

**TABLE 4:** Total and differential leukocytes counts (10^9^ cells/L) in higher‐(poly)phenol (H‐POL) and lower‐(poly)phenol (L‐POL) conditions.

	Pre‐exercise	Post‐exercise	1‐h post‐exercise	2‐h post‐exercise	Time		Condition		Interaction	
					*P* value	η_p_^2^	*P* value	η_p_^2^	*P* value	η_p_^2^
Leukocytes										
H‐POL	5.96 ± 1.29	9.85 ± 1.90*	6.45 ± 1.69	7.79 ± 2.26*	**<0.001**	0.71	0.758	0.01	0.537	0.03
L‐POL	5.80 ± 1.26	9.72 ± 2.73*	7.04 ± 1.72	8.30 ± 2.13*						
Neutrophils										
H‐POL	2.87 ± 0.67	4.23 ± 0.99*	3.89 ± 1.50*	5.01 ± 2.06*	**<0.001**	0.61	0.281	0.05	0.393	0.04
L‐POL	3.04 ± 1.15	4.72 ± 1.67*	4.69 ± 1.77*	5.84 ± 2.11*						
Monocytes										
H‐POL	0.51 ± 0.17	0.81 ± 0.32*	0.49 ± 0.22	0.59 ± 0.21	**<0.001**	0.52	0.838	0.01	0.746	0.02
L‐POL	0.47 ± 0.17	0.82 ± 0.36*	0.52 ± 0.17	0.54 ± 0.26						
Basophils										
H‐POL	0.08 ± 0.02	0.12 ± 0.05*	0.07 ± 0.02	0.08 ± 0.02	**<0.001**	0.44	0.400	0.03	0.934	0.00
L‐POL	0.07 ± 0.01	0.11 ± 0.03*	0.07 ± 0.01	0.07 ± 0.01						
Eosinophils										
H‐POL	0.24 ± 0.15	0.29 ± 0.22*	0.19 ± 0.09†	0.17 ± 0.09†	**<0.001**	0.37	0.163	0.08	0.374	0.05
L‐POL	0.16 ± 0.17	0.17 ± 0.18*	0.12 ± 0.16†	0.07 ± 0.04†						
Platelets										
H‐POL	257 ± 32	309 ± 73*	261 ± 37	270 ± 43	**<0.001**	0.40	**0.025**	0.19	0.773	0.02
L‐POL	209 ± 37	279 ± 0.56*	230 ± 0.38	229 ± 052						
NL ratio										
H‐POL	1.40 ± 0.39	1.05 ± 0.34	2.32 ± 0.93*	2.78 ± 1.18*	**<0.001**	0.68	0.124	0.09	0.124	0.08
L‐POL	1.52 ± 0.64	1.37 ± 0.76	3.20 ± 1.82*	3.52 ± 1.43*						
PL ratio										
H‐POL	127 ± 36	75 ± 21†	157 ± 37*	150 ± 40*	**<0.001**	0.73	0.584	0.01	0.137	0.08
L‐POL	105 ± 21	81 ± 32†	151 ± 48*	139 ± 48*						
SII										
H‐POL	360 ± 111	323 ± 128	612 ± 257*	763 ± 372*	**<0.001**	0.58	0.419	0.01	0.515	0.04
L‐POL	328 ± 178	384 ± 222	744 ± 496*	857 ± 533*						

Values are means ± SD; all *n* = 13 per condition unless stated otherwise. *Increased above pre‐exercise values (*post hoc*); †Decreased below pre‐exercise values (*post hoc*). Basophils: *n* = 12 H‐POL, *n* = 13 L‐POL; eosinophils: *n* = 12 H‐POL, *n* = 13 L‐POL. Analysis performed with linear mixed models. *P*‐values shown in bold are considered statistically significant. NL, neutrophil–lymphocyte; PL, platelet lymphocyte; SII, systemic inflammatory index; WBC, white blood cells.

### 3.4 Cytokines, chemokines and growth factors

Data and results from the main LMM analysis are presented in Table 5. IL‐6 increased 2 h post‐exercise (*P* = 0.017) and IL‐10 increased 1 h post‐exercise (*P* < 0.001). There were interaction effects for IL‐10 and TNF‐α; however, *post hoc* adjusted tests indicated no significant differences at any time points. IFN‐γ decreased post‐exercise (*P* = 0.049), 1 h (*P* < 0.001) and 2 h post‐exercise (*P* < 0.001). MCP‐1 increased post‐exercise (*P* = 0.033) and 1 h post exercise (*P* < 0.001). There was a main time effect for GCSF (*P* = 0.037); however, there were no significant differences with *post hoc* tests.

**TABLE 5:** Serum cytokine, growth factor, chemokine and oxidative stress marker response before (pre), after (post), at 1 h and 2 h post‐exercise in higher‐(poly)phenol (H‐POL) and lower‐(poly)phenol (L‐POL) conditions.

Biomarkers	Pre‐exercise	Post‐exercise	1‐hr post exercise	2‐hr post exercise	Time		Condition		Interaction	
					*P*	η_p_^2^	*P*	η_p_^2^	*P*	η_p_^2^
IL‐6 (pg/mL)										
H‐POL	1.24 ± 1.08	1.46 ± 0.75	1.66 ± 0.81	2.36 ± 2.25*	**0.018**	0.13	0.599	0.01	0.889	0.01
L‐POL	1.42 ± 1.03	1.62 ± 1.31	2.12 ± 1.02	2.32 ± 1.03*						
IL‐10 (pg/mL)										
H‐POL	2.37 ± 1.73	2.33 ± 1.12	2.65 ± 1.39	1.48 ± 0.47	**<0.001**	0.46	0.822	0.01	**0.001**	0.21
L‐POL	1.80 ± 0.66	1.88 ± 0.82	3.22 ± 1.02	1.82 ± 0.76						
TNF‐α (pg/mL)										
H‐POL	7.06 ± 1.86	7.23 ± 1.64	6.54 ± 1.47	6.73 ± 1.51	0.604	0.02	0.992	0.04	**0.043**	0.11
L‐POL	6.55 ± 1.07	6.74 ± 1.44	7.41 ± 2.35	6.84 ± 1.01						
IFN‐γ (pg/mL)										
H‐POL	0.96 ± 1.14	0.83 ± 0.98†	0.76 ± 0.77†	0.43 ± 0.22†	**<0.001**	0.31	0.869	0.00	0.964	0.00
L‐POL	0.88 ± 0.94	0.79 ± 0.9†	0.71 ± 0.68†	0.69 ± 0.85†						
MCP‐1 (pg/mL)										
H‐POL	143 ± 25	163 ± 30*	166 ± 31*	144 ± 30	**<0.001**	0.32	0.809	0.00	0.280	0.05
L‐POL	141 ± 19	155 ± 23*	182 ± 40*	145 ± 22						
G‐CSF (pg/mL)										
H‐POL	16 ± 6	19 ± 6	18 ± 7	20 ± 6	**0.037**	0.12	0.902	0.00	0.842	0.01
L‐POL	16 ± 9	18 ± 8	18 ± 8	16 ± 5						
8‐OHdG (ng/mL creatinine)										
H‐POL	14.3 ± 22.9	7.7 ± 9.5	8.5 ± 8.9	6.8 ± 5.4	0.278	0.05	**<0.001**	0.51	0.113	0.08
L‐POL	1.35 ± 0.51	1.51 ± 0.72	2.24 ± 1.23	3.19 ± 2.5						
GPX (nmol/min/mL)										
H‐POL	1429 ± 632	1341 ± 700	1294 ± 473	1541 ± 679	0.920	0.01	**<0.001**	0.39	0.835	0.01
L‐POL	2192 ± 537	2348 ± 743	2296 ± 598	2322 ± 923						
4‐HNE (pg/mL)										
H‐POL	6.8 ± 2.9	6.2 ± 3.1	8.4 ± 4.6	12.9 ± 16.1	0.384	0.07	0.466	0.03	0.582	0.04
L‐POL	5.8 ± 3.9	7.9 ± 8.2	8.6 ± 6.5	9.5 ± 10.3						

Values are means ± SD; all *n* = 13 per condition unless stated otherwise. *Increased above pre‐exercise values (*post hoc*); †decreased below pre‐exercise values (*post hoc*). Analysis performed with linear mixed models. *P*‐values shown in bold are considered statistically significant. 4‐HNE, 4‐hydroxy‐2‐nonenal (*n* = 7 L‐POL, *n* = 11 H‐POL); 8‐OHdG, 8‐hydroxy‐ 2′‐deoxyguanosine (*n* = 13 H‐POL, *n* = 12 L‐POL); G‐CSF, granulocyte colony‐stimulating factor; GPX, glutathione peroxidase (*n* = 12 H‐POL, *n* = 11 L‐POL); IFN‐γ = interferon‐γ; IL‐6, interleukin 6; IL‐10 = interleukin 10; MCP‐1, monocyte chemoattractant protein‐1; TNF‐α, tumour necrosis factor‐α.

#### 3.4.1 Oxidative stress markers

There were condition effects for 8‐OHdG and GPX concentrations; the former was significantly higher, whilst the latter was significantly lower in the H‐POL condition (*P* < 0.001). As shown in Table 5, there was marked inter‐individual variability in 8‐OHdG concentrations in the H‐POL condition, especially pre‐exercise. Therefore, to check the robustness of these findings, data from three participants who had pre‐exercise concentrations >20 ng/mL creatinine were removed for a sensitivity analysis. We used a 20 ng/mL creatinine cut‐off as this was the mean upper range reported in healthy adults in a meta‐analysis of urinary 8‐OHdG concentrations (Graille et al., 2020). This reduced pre‐exercise levels to 4.21 ± 2.9 ng/mL, but as the condition effect remained with data from these three participants removed (*P* < 0.001), and we did not have a reasonable justification to remove these as outliers, the original data were analysed and presented.

There were no main effects for plasma 4‐HNE concentrations. Note, several samples were below the detectable level, so 4‐HNE data were only analysed for seven participants in L‐POL and 11 participants in H‐POL.

#### 3.4.2 Correlation analysis

The change in total (poly)phenol content at 1 h post‐exercise was negatively correlated with the change in IL‐10 at the same time point (rho −0.465; *P* = 0.018). There were no other correlations between total (poly)phenol content and the other outcomes.

## 4 DISCUSSION

This study investigated the effects of a (poly)phenol‐rich food intervention on markers of inflammation and oxidative stress following exercise. The key findings indicated that consuming a H‐POL food intervention significantly increased total plasma (poly)phenol concentration but did not significantly modulate several markers of post‐exercise inflammation. However, GPX activity was significantly higher in the L‐POL condition, whereas urinary 8‐OHdG excretion was elevated in the H‐POL condition (condition effects). Taken together, these results suggest that short‐term intake of a H‐POL food intervention before exercise did not significantly alter inflammatory markers but may have modified markers of oxidative stress, albeit with some inconsistent effects.

(Poly)phenols, whether consumed in solid or liquid form, are only present in the bloodstream for a limited period before being excreted (Murray et al., 2021; Selby‐Pham et al., 2018). These bioactive compounds are absorbed passively in the small intestine, attain peak concentrations in the circulation 1–4 h post‐intake, and are largely eliminated within 8 h, indicating a restricted time frame for their biological activity (Corona et al., 2016; Lee et al., 2002; Murray et al., 2021; Selby‐Pham et al., 2018). In this study, there was no significant difference between conditions in total (poly)phenol plasma concentration pre‐exercise (after 3 days of the interventions), indicating that they were eliminated from the circulation sometime in the ∼24 h after they were last consumed. In contrast, flavonols, hydroxybenzoic acids and tyrosyls, various individual metabolites, and total (poly)phenol concentrations, were significantly elevated approximately 1 and 2 h after ingestion (post‐exercise and 1 h post‐exercise, respectively). These results align with previous research demonstrating that plasma concentrations of total (poly)phenols or individual metabolites after consuming (poly)phenol‐enriched food or supplements are elevated 1 and 4 h post‐intake (Cheok et al., 2022; Corona et al., 2016; Lee et al., 2002; Zhong et al., 2017).

While the H‐POL intervention significantly increased plasma (poly)phenol concentrations, there were no marked condition differences in the inflammatory markers, nor a consistent pattern of change favouring one condition. Platelets were higher in the H‐POL condition (condition effect; η_p_^2^ = 0.19) and there were some cytokines (IL‐10 and TNF‐α) that showed interaction effects (both higher 1 ‐ 2h post‐exercise in L‐POL; η_p_^2^ ≥ 0.11), but neither was statistically significant with *post hoc* tests, suggesting the effects were small and unlikely meaningful. In addition, this could also be due to insufficient statistical power to detect these effects. Given the general lack of differences between the conditions, it is perhaps unsurprising that there were no significant correlations between the change in plasma total (poly)phenol metabolites and the change in the inflammatory markers measured, other than for IL‐10; thus, limited mechanistic insights can be gleaned from these analyses. These findings somewhat contrast with an earlier study that showed an antioxidant‐rich food diet reduced some markers of inflammation across a 3‐week training camp in young, trained athletes (Koivisto et al., 2019). Indeed, Koivisto et al. (2019) found that incorporating antioxidant‐rich foods (21.2 mmol/day) such as vegetables, fruits, dried or as smoothies, nuts, and dark chocolate markedly lowered levels of CRP, IL‐13 (while increased in control) and IL‐6 during and after an intense training period, compared to a control group consuming a lower antioxidant diet (2.8 mmol/day). However, akin to our study, there were no significant between‐condition differences reported for several other cytokines and chemokines. Our results also contrast with those of Plunkett et al. (2010), who found that restricting foods known to be rich in antioxidants for 2 weeks was associated with higher circulatory TNF‐α levels post exhaustive exercise. However, neither of these studies and, to our knowledge, no previous studies, have quantified total (poly)phenol concentrations alongside markers of inflammation post‐exercise. The discrepancy in findings between these studies and ours could be partly due to the shorter duration of our intervention, which was perhaps insufficient to markedly alter endogenous inflammatory or antioxidant pathways that regulate the markers measured. It is also possible that the dose provided in the present study, which is likely lower than that provided in most (poly)phenol‐based supplements (Bowtell & Kelly, 2019; Rickards et al., 2021), was not sufficient to modulate systemic changes in inflammation or oxidative stress. However, it remains unclear what the best dose or duration of intake is to modify inflammatory status, and whether larger or longer‐term intakes are needed to induce biochemical changes that may reinforce host defences to stresses, such as the upregulation of Nrf2 (Clifford et al., 2021b). Our understanding of the optimal dose and duration is currently limited by the lack of studies that quantify the (poly)phenol content of the food interventions and, importantly, their subsequent bioavailability. However, our study suggests that the 285 mg of (poly)phenols provided, and ∼240,000 nM concentrations of plasma (poly)phenols reached post‐ingestion, were maybe not sufficient to markedly alter post‐exercise markers of systemic inflammation.

This study also measured plasma 4‐HNE, erythrocyte GPX activity and urinary excretion of 8‐OHdG as markers of oxidative stress. Interestingly, erythrocyte GPX activity was significantly lower in the H‐POL vs. L‐POL intervention at all time points. These findings agree with a previous study in military personnel where erythrocyte GPX activity was significantly lower after consuming resveratrol (100 mg/day for 90 days) compared to a placebo after a fitness test (Macedo et al., 2015). However, results with (poly)phenol interventions are mixed, and whilst not in erythrocytes, one study reported no effects of curcumin (180 mg) on plasma GPX activity <2 h post‐exercise (Takahashi et al., 2013), while another study reported that pomegranate juice (500 mL) increased plasma GPX activity immediately after weight lifting exercise (Ammar et al., 2017). Nonetheless, changes in GPX activity are difficult to interpret in isolation. While the higher GPX activity in the L‐POL condition suggests higher activation of this antioxidant enzyme, this could imply either a counteractive response to heightened reactive oxidants/oxidative stress, or boosted protection against reactive oxidant‐induced damage (Jackson, 1999). The former is at odds with the lower urinary 8‐OHdG in the L‐POL intervention, which could reflect lower DNA‐related oxidative damage, suggesting oxidative stress was not elevated in the L‐POL condition but possibly higher in the H‐POL condition. However, it should be noted that while there were condition effects for greater 8‐OHdG levels, and lower GPX activity in the H‐POL condition, there were no statistically significant interaction effects. Moreover, pre‐exercise values for both GPX and 8‐OHdG were markedly different between conditions; indeed, the values for 8‐OHdG were markedly higher in H‐POL, and more variable, especially pre‐exercise. Taken together, this suggests the condition differences were partly driven by the markedly different pre‐exercise values. Given that there were no time effects for an increase in markers of oxidative stress, and differences in plasma (poly)phenol levels pre‐exercise, or any correlations between (poly)phenol concentrations and these markers, it seems unlikely that the pre‐exercise differences in GPX and 8‐OHdG were directly related to the H‐POL intervention; thus, pro‐oxidant effects seem unlikely. Instead, these results could be due to random chance or partly driven by the unexplainable but much greater inter‐individual variability in the H‐POL condition, as shown in Table 5. Therefore, pending further studies, these results should be interpreted cautiously.

A key strength of this study is the quantification of a comprehensive array of total plasma (poly)phenol metabolites following a mixed (poly)phenol‐rich meal before and after exercise in humans. We are unaware of any previous studies examining the effects of (poly)phenol foods on exercise‐induced biochemical changes that have reported the plasma (poly)phenol concentrations alongside their outcomes. Furthermore, this study measured a wider array of inflammatory markers than most previous studies assessing the impact of (poly)phenol interventions on post‐exercise inflammation (Doma et al., 2020; Rickards et al., 2021). However, there are some limitations that warrant consideration. Some of the cytokines (IL‐1β, IL‐2, IL‐4, IL‐12) were under the limits of detection and could not be quantified. Although many previous studies have also not detected these cytokines post‐exercise (Hirose et al., 2004; Suzuki et al., 2000), we had hoped the new more sensitive technology we use would overcome this limitation. Future research should consider omitting these cytokines and focusing on others suggested to modulate the post‐exercise inflammatory response. The measurement of oxidative stress markers was limited in scope due to resource constraints; hence, these were secondary outcomes in this study. Additionally, to reduce participant burden we did not collect a blood sample before the 3‐day pre load, meaning we are unable to determine if the intervention modified pre‐exercise values. We also did not collect samples at 24–96 h post‐exercise when muscle damage may have been present, and group differences were apparent. Lastly, all markers were measured systemically or in urine, and therefore we cannot rule out changes in skeletal muscle or other tissues.

### 4.1 Conclusion

Short‐term consumption of a higher‐(poly)phenol food intervention increased total plasma (poly)phenol concentrations but did not significantly modify markers of inflammation and had inconsistent and unclear effects on markers of oxidative stress 0–2 h following strenuous exercise. Overall, there was no clear pattern of change to suggest that the large increase in plasma (poly)phenol concentrations could reduce post‐exercise changes in markers of oxidative stress and inflammation, which have been associated with delayed exercise recovery and immune suppression. Future research, possibly with higher (poly)phenol doses and markers in different tissues, are needed to determine if (poly)phenol‐rich meals are able to modify the exercise‐induced inflammatory and oxidative stress response.

## AUTHOR CONTRIBUTIONS

The research was carried out at the National Institute for Health and Care Research (NIHR) Leicester Biomedical Research Centre (BRC). Conceptualization (Tom Clifford, Abrar Al Hebshi, Lewis J James, Josh Thorley). Collection, analysis, or interpretation of data (Tom Clifford, Abrar Al Hebshi, Josh Thorley, Zicheng Zhang, Ana Rodriguez‐Mateos). Writing—original draft preparation (Tom Clifford, Abrar Al Hebshi, Zicheng Zhang). Writing—review and editing (Tom Clifford, Abrar Al Hebshi, Lewis J James, Josh Thorley, Zicheng Zhang, Ana Rodriguez‐Mateos). All authors have read and approved the final version of this manuscript and agree to be accountable for all aspects of the work in ensuring that questions related to the accuracy or integrity of any part of the work are appropriately investigated and resolved. All persons designated as authors qualify for authorship, and all those who qualify for authorship are listed.

## CONFLICT OF INTEREST

The authors declare no conflicts of interest for this study.

## FUNDING INFORMATION

No funding was received for this work. L.J.J is funded by the NIHR Leicester BRC.

## Data Availability

The datasets used and/or analysed during the current study may be available from the corresponding author on reasonable request. Individual participant data cannot be shared publicly due to ethical constraints. The aggregate data supporting the conclusions of the manuscript are presented in the manuscript.

## 1 Quantification of (poly)phenols in dietary interventions

Flavan‐3‐ols (monomers and procyanidins) in approximately 5 g of food samples (or 10 g of juice samples) were extracted with a total of 3 mL of acetone–water–acetic acid (70:29.5:0.5, v/v) by vortexing (5 min), sonicating (15 min) at 50°C, vortexing again (2 min) and centrifugating at 1800 *g* (15 min). Supernatants were pooled and purified using Strata SCX SPE cartridges (Phenomenex, CA, USA) (500 mg/3 mL, 55 µm, 70 Å), which were pre‐conditioned with 5 mL of water. The first millilitre of eluate was discarded, while the remaining 2 mL was collected and further filtered through 0.22 µm filters before transferring to HPLC amber vials.

The quantification of flavan‐3‐ols (monomers and procyanidins) was performed based on the method previously described using HPLC with a fluorescence detector (HPLC‐FLD) (Bussy et al., 2020; Rodriguez‐Mateos et al., 2012). Five microlitres of flavan‐3‐ols were separated by an Agilent (Santa Clara, CA, USA) 1100 series HPLC system equipped with a fluorescence detector and a Torus Diol column (100 × 3.0 mm, 130 Å, 5.0 µm) under 40°C. Mobile phases were acetonitrile–acetic acid (98:2, v/v) as solvent A and methanol–water–acetic acid (95:3:2, v/v) as solvent B. The gradients were as follows: 0–3 min, 0% B; 3–15 min, 0–10% B; 15–25 min, 10–30% B; 25–37 min, 30–45% B; 37–40 min, 45–95% B; 40–45 min, 95% B; and 45–50 min, 95–0% B, with a flow rate of 0.3 mL/min. The fluorescence detection was conducted with an excitation of 230 nm and emission at 321 nm. Calibration curves were obtained using NIST cocoa extract RM 8403 standard.

Anthocyanidins, flavonols, and other (poly)phenols in approximately 1 g of food samples (or 5 g of juice samples) were extracted using acidified methanol (0.1 % formic acid, v/v) by vortexing (5 min), sonicating (15 min) and centrifugating at 1800 *g* (15 min). Supernatants were pooled with the final volume of 25 mL for various analyses.

Quantification of anthocyanidins was performed based on the method previously described (Rodriguez‐Mateos et al., 2012) using HPLC with with diode array detection (HPLC‐DAD). Three millilitres of supernatants were mixed with 3 mL of 5 M HCl and incubated in a water bath under 90°C for 1 h to hydrolyse and cleave anthocyanins into anthocyanidins. All samples were then filtered through 0.22 µm filters and transferred to HPLC amber vials. Ten microlitres of anthocyanidins were separated by an Agilent 1100 series HPLC system equipped with a diode array detector and a Poroshell 120 EC‐C18 column (100 × 2.1 mm, 2.7 µm) under 35°C. Mobile phases were methanol–water–5 M HCl (5:94.9:0.1, v/v/v) as solvent A and acetonitrile–water–5 M HCl (50:49.9:0.1, v/v/v) as solvent B. The gradients were as follows: 0–2 min, 5% B; 2–20 min, 5–50% B; 20–30 min, 50–95% B; 30–35 min, 95 % B; 35–40 min, 95–5% B, with a flow rate of 0.5 mL/min. The eluate was monitored at 520 nm, and calibration curves were obtained using anthocyanin standards after the same acid hydrolysis procedure.

Purification of flavonols and other (poly)phenols in food samples was performed using solid phase extraction (SPE). Three millilitres of supernatant was acidified with 4% phosphoric acid (1:1, v/v), and the mixture was then loaded onto an Oasis PRiME hydrophilic−lipophilic balanced SPE cartridges (Waters Limited, Wilmslow, UK) (3 mL, 150 mg). Cartridges were washed with acidified water (0.1% formic acid, v/v) and targeted compounds were eluted with acidified methanol (0.1% formic acid, v/v), filtered (0.22 µm) and transferred to HPLC amber vials.

Quantification of flavonols in food samples was achieved using UPLC–ESI–QqQ–MS/MS. Five microlitres of sample was injected into the system and passed through a Poroshell 120 EC‐C18 column (100 × 2.1 mm, 2.7 µm) coupled with a compatible EC‐C18 guard cartridge (5 × 2.1 mm, 2.7 µm) under 25°C. Mobile phases were HPLC grade water and acetonitrile that were both acidified with 0.1% formic acid (v/v), as solvent A and B, respectively. The gradients were as followed: 0–0.2 min, 5% B; 0.2–2 min, 5–17% B; 2–10 min, 17–19.5% B; 10–10.5 min, 19.5–50% B; 10.5–11 min, 50–90% B; 11–13 min, 90%B; 13–13.5 min, 90–5%B; and 13.5–16 min, 5% B, with a flow rate of 0.4 mL/min by the ACCELA quaternary pump (Thermo Fisher Scientific, MA, USA). The MS analysis was performed under the following conditions: collision gas pressure 1.5 mTorr, capillary temperature 300°C, vaporizer temperature 350°C, sheath gas pressure 40 arb, aux gas pressure 10 arb, spray voltage 2500 V. All of the compounds were analysed in negative ion mode. Due to the shortage of the commercial flavonols and the corresponding derivative standards, eight HPLC grade standards were used for the identification and quantification of the compounds. All standards were mixed as the master calibration mixture, and eight dilutions were made for plotting the calibration curve. Taxifolin (0.25 mg/mL) was fortified into all samples and calibration mixes in the same concentration as the internal standard. The LC‐MS parameters of those compounds that lack commercial standards were obtained from published references (Alvarez‐Fernandez et al., 2015; Pertuzatti et al., 2021; Renai et al., 2021; Vrhovsek et al., 2012) and later verified according to the results of optimisation on the full sample pool. The final MRM analysis method was generated with the confirmed retention time of the compounds, 2 min RT window, 0.70 FWHM Q1 peak width, and 3.0 s cycle time. The peak integration was conducted with TraceFinder 5.0 Software (Thermo Fisher Scientific, Runcorn, UK) and data calculation was performed in Microsoft Excel. The area ratios of the target compounds to internal standard taxifolin were used in the quantification to balance the variations in device performance during the batch run. Quantification of other (poly)phenols in food samples was performed using UPLC‐ESI‐QqQ‐MS/MS based on the method described in Methods, ‘Quantification for (poly)phenol metabolites’.

**TABLE A1:** List of (poly)phenols measured in the two dietary interventions.

Procyanidin
Delphinidin
Cyanidin
Petunidin
Pelargonidin
Peonidin
Malvidin
2,3‐Dihydroxybenzoic acid
2,4‐Dihydroxybenzoic acid
2,5‐Dihydroxybenzoic acid
2,6‐Dihydroxybenzoic acid
2‐Hydroxybenzoic acid
3‐(4‐Hydroxy‐3‐methoxyphenyl)propionic acid
3,4‐Dihidroxyphenylacetic acid
3,4‐Dihydroxycinnamic acid
3,5‐Dihydroxybenzoic acid
3‐Feruloylquinic acid
4‐Caffeoylquinic acid
4‐Feruloylquinic acid
4‐Hydroxybenzaldehyde
4‐Hydroxybenzoic acid
Caffeic acid
Caffeine
Catechin
Chlorogenic acid
Epicatechin
Ethyl gallate
flavone
Gallic acid
Genistein
Hesperetin
Isoferulic acid
Kaempferol
L‐Tartaric acid
*m*‐Coumaric acid
Morin
Myricetin
*o*‐Coumaric acid
*p*‐Coumaric acid
Phloretin
Procyanidin A2
Protocatechuic acid
Pyrogallol
Quercetin
Secoisolariciresinol
Syringic acid
Theobromine
Theophylline
*trans*‐Ferulic acid
Trigonelline
Tyrosol
Vanillic acid
Vanillin
Quercetin‐3‐rhamnoside
Quercetin‐3‐rhamnoside‐2
Quercetin‐3‐hexoside‐1
Quercetin‐3‐hexoside‐2
Quercetin‐3‐hexoside‐3
Quercetin‐3‐hexoside‐4
Quercetin‐3‐aldopentoside‐1
Quercetin‐3‐aldopentoside‐2
Quercetin‐3‐aldopentoside‐3
Quercetin‐3‐rutinoside
Quercetin‐3‐acetyl‐hexoside
Quercetin‐3‐deoxyhexose‐hexoside
Kaempferol‐3‐aldopentoside
Kaempferol‐3‐hexoside‐1
Kaempferol‐3‐hexoside‐2
Kaempferol‐3‐rutinoside
Kaempferol‐3‐rutinoside‐2
Kaempferol‐3‐rutinoside‐3
Isorhamnetin‐3‐glucoside
Isorhamnetin‐3‐hexoside‐1
Isorhamnetin‐3‐hexoside‐2
Isorhamnetin‐3‐aldopentoside‐1
Isorhamnetin‐3‐aldopentoside‐2
Isorhamnetin‐3‐rutinoside
Isorhamnetin‐3‐acetyl‐hexoside
Myricetin‐3‐rhamnoside
Myricetin‐3‐aldopentoside
Myricetin‐3‐hexoside‐1
Myricetin‐3‐hexoside‐2
syringetin‐3‐hexoside‐1
Laricitrin‐3‐hexoside
4‐Methylhippuric acid
Kaempferol‐7‐rhamnoside
Laricitrin‐3‐aldopentoside
Laricitrin‐3‐rhamnoside
Laricitrin‐3‐acetyl‐hexoside
Syringetin‐3‐acetyl‐hexoside
Syringetin‐3‐rhamnoside
Phloretin‐2‐glucoside
Myricetin‐3‐acetyl‐hexoside
Methyl gallate
Caffeic acid hexoside
Rhamnetin
Isorhamnetin
Laricitrin

**TABLE A2:** Plasma (poly)phenol metabolites measured.

**Cinnamic acids**	
4‐Feruloylquinic acid 3,4‐Dihidroxyphenylacetic acid Caffeic acid 3‐*O*‐sulfate Caffeic acid 4‐*O*‐sulfate *p*‐Coumaric acid‐4‐*O*‐β‐d‐glucuronide 3,4‐Dihydroxycinnamic acid Chlorogenic acid Dihydro isoferulic acid 3‐*O*‐sulfate Isoferulic acid 3‐*O*‐sulfate Isoferulic Acid 3‐*O*‐β‐d‐glucuronide *p*‐Coumaric acid 3‐Feruloylquinic acid Phenylacetic acid Sinapic acid Caffeic acid‐4‐glucuronide *o*‐Coumaric acid	*p*‐Coumaric acid 4‐*O*‐sulfate dissodium salt Caffeic acid‐3‐glucuronide Dihydro ferulic acid 4‐*O*‐sulfate Ferulic acid 4‐*O*‐sulfate disodium salt *trans*‐Ferulic acid Caffeic acid 4‐Caffeoylquinic acid Dihydro ferulic acid 4‐*O*‐β‐d‐glucuronide Dihydro caffeic acid 3‐*O*‐sulfate sodium salt Ferulic acid 4‐*O*‐β‐d‐glucuronide 4‐Feruloylquinic acid Isoferulic acid *m*‐Coumaric acid
**Phenylpropanoic acids**	
3‐(4‐Hydroxy‐3‐methoxyphenyl)propionic acid dl‐*p*‐Hydroxyphenyllactic acid 3‐(3‐Hydroxyphenyl)propanoic acid 3‐(2‐Hydroxyphenyl) propionic acid 4‐Hydroxyphenylpropionic acid	3‐(2,3‐Dihydroxyphenyl)propionic acid 3‐(4‐Hydroxy‐3‐methoxyphenyl)propionic acid Dihydro isoferulic acid 3‐*O*‐?‐d‐glucuronide
**Benzene diols and triols**	
Pyrogallo‐1‐*O*‐sulfate Pyrogallo‐2‐*O*‐sulfatee 2‐Methylpyrogallol‐*O*‐sulfate 4‐Methylcatechol‐1‐sulfate Vanillin	1‐Methylpyrogallol‐*O*‐sulfate 4‐Methylcatechol Catechol‐*O*‐1‐glucuronide
**Hydroxybenzoic acids**	
Gallic acid Protocatechuic acid 3‐*O*‐sulfate Protocatechuic acid 4‐*O*‐sulfate 2,3‐Dihydroxybenzoic acid 2,5‐Dihydroxybenzoic acid Isovanillic acid 3‐*O*‐sulfate Homovanillic acid sulfate sodium salt 2,6‐Dihydroxybenzoic acid 3‐Hydroxybenzoic acid 2‐Hydroxy‐4‐methoxybenzoic acid Ethyl gallate	4‐Methylgallic‐3‐*O*‐sulfate 3,5‐Dihydroxybenzoic acid Vanillic acid‐4‐*O*‐sulfate Protocatechuic acid 3‐*O*‐β‐d‐glucuronide 2‐Hydroxybenzoic acid 2,4‐Dihydroxybenzoic acid Protocatechuic acid Vanillic acid 4‐Hydroxybenzoic acid 2,3,4‐Trihydroxybenzoic acid
**Hippuric acids**	
4‐Hydroxyhippuric acid 3‐Hydrxyhippuric acid α‐Hydroxyhippuric acid	Hippuric acid 2‐Hydroxyhippuric acid
**Tyrosols**	
Hydroxytyrosol Hydroxy tyrosol 4‐sulfate sodium salt Hydroxy tyrosol 3‐sulfate sodium salt Tyrosol sulfate sodium salt	(4*R*)‐5‐(3′,4′‐Dihydroxyphenyl)‐gamma‐ valerolactone‐4′‐*O*‐sulfate sodium salt Tyrosol
**Caffine metabolites**	
1‐Methylxanthine Theophylline	3‐Methylxanthine
**Dihydrocaffeic acids**	
Dihydrocaffeic acid‐3‐*O*‐β‐d‐glucuronide	
**Benzaldehydes**	
4‐Hydroxybenzaldehyde 3,4‐Dihydroxybenzaldehyde	
**Flavonols**	
(‐)‐Epicatechin‐3‐*O*‐sulfate Epicatechin Quercetin 3‐sulfate potassium salt (−)‐Epicatechin‐3‐*O*‐methylether Procyanidin A2 Myricetin Kaempferol	Quercetin 3‐*O*‐β‐d‐glucuronide (−)‐Epicatechin‐4‐*O*‐methylether Quercetin‐7‐*O*‐β‐d‐glucuronide Kaempferol‐3‐glucuronide Morin Quercetin
**Hydroxycoumarins**	
7,8‐Dihydroxycoumarin Isourolithin A	Urolithin B Urolithin A
**Stilbenes**	
*trans*‐Resveratrol 4′‐*O*‐β‐d‐glucoronide *trans*‐Resveratrol 3‐*O*‐β‐d‐glucuronide *trans*‐Resveratrol 3‐sulfate sodium salt	*cis*‐Resveratrol 4′‐*O*‐β‐d‐glucuronide *cis*‐Resveratrol 3‐*O*‐β‐d‐glucuronide
**Lignans**	
SECO Enterodiol Enterolatone glucuroinde 6	Enterolatone Enterolatone sulfate
**Isoflavonoids**	
Equol glucuronide Equol sulfate	Daidzein Phloretin
**Flavonones**	
Naringenin 4‐*o*‐glucurionide Naringenin 7‐*o*‐glucurionide	*rac*‐Hesperetin 7‐*O*‐sulfate sodium salt Hesperetin
**Aryloxyalkanoic Acids**	
(*R*)‐(+)‐2‐(4‐Hydroxyphenoxy)‐propionic acid	

**TABLE A3:** *Post hoc P*‐values at post‐exercise and 1 h post‐exercise in the in higher‐(poly)phenol (H‐POL) and lower‐(poly)phenol (L‐POL) conditions for individual (poly)phenol metabolites that showed significant main interaction and/or condition effects.

Plasma (poly)phenol metabolite	Post‐exercise	1‐h post‐exercise
(‐)‐Epicatechin‐3‐*O*‐sulfate	**<0.001**	**<0.001**
Tyrosol sulfate sodium salt	**<0.001**	**<0.001**
(4*R*)‐5‐(3′,4′‐Dihydroxyphenyl)‐γ‐valerolactone‐4′‐*O*‐sulfate sodium salt	0.081	0.128
Gallic acid	**0.012**	**0.008**
Protocatechuic acid 3‐*O*‐sulfate	**<0.001**	**<0.001**
Protocatechuic acid 4‐*O*‐sulfate	**<0.001**	**<0.001**
4‐Methylgallic‐3‐*O*‐sulfate	**<0.001**	**<0.001**
Isovanillic acid 3‐*O*‐sulfate	**0.033**	0.350

*P*‐values shown in bold are considered statistically significant.
